# Sustaining alcohol and opioid use disorder treatment in primary care: a mixed methods study

**DOI:** 10.1186/s13012-018-0777-y

**Published:** 2018-06-18

**Authors:** Sarah B. Hunter, Allison J. Ober, Colleen M. McCullough, Erik D. Storholm, Praise O. Iyiewuare, Chau Pham, Katherine E. Watkins

**Affiliations:** RAND Corporation, 1776 Main Street, Santa Monica, CA 90407-2138 USA

**Keywords:** Behavioral health care integration, Sustainment, Mixed methods

## Abstract

**Background:**

Efforts to integrate substance use disorder treatment into primary care settings are growing. Little is known about how well primary care settings can sustain treatment delivery to address substance use following the end of implementation support.

**Methods:**

Data from two clinics operated by one multi-site federally qualified health center (FQHC) in the US, including administrative data, staff surveys, interviews, and focus groups, were used to gather information about changes in organizational capacity related to alcohol and opioid use disorder (AOUD) treatment delivery during and after a multi-year implementation intervention was executed. Treatment practices from the intervention period were compared to practices after the intervention period to examine whether the practices were sustained. Data from staff surveys and interviews were used to examine the factors related to sustainment.

**Results:**

The two clinics sustained multiple components of AOUD care 1 year following the end of implementation support, including care coordination, psychotherapy, and medication-assisted treatment. Some of the practices were modified over time, for example, screening became less frequent by design, while use of care coordination and psychotherapy for AOUDs expanded. Participants identified staff training and funding for medications as key challenges to sustaining treatment.

**Conclusions:**

Following a multi-year implementation intervention, a large FQHC continued to deliver AOUD treatment. Access to external funding and staff support appeared to be critical elements for sustaining care over time.

**Trial registration:**

clinicaltrials.gov identifier: NCT01810159

## Background

Alcohol and opioid use disorders are pervasive public health problems that are frequently under-identified and untreated. An estimated 15.1 million people suffer from an alcohol use disorder in the United States (US) and an estimated 4.8 million misuse opioids [1]. The consequences of alcohol and opioid use disorders include increased risk of disease, injury, disability, and death [2, 3]. Furthermore, the societal costs for each of these disorders are estimated to be several billions annually [4, 5]. Nevertheless, only a small fraction of people in need of treatment for alcohol or opioid misuse access it in any given year [6]. Research suggests that limited availability, lack of insurance coverage, waitlists, and stigma prevent those in need of specialty substance use treatment from accessing it [7].

Efforts are underway to integrate substance use disorder treatment into primary care to increase access to treatment for the millions of people who never receive it in specialty care [8, 9]. Health care coverage changes in the US have supported the provision of behavioral health care in general medical settings [10, 11]. In addition, treatments for substance use disorders, including medication-assisted treatment for alcohol and opioid use disorders (AOUDs), have been shown to be effective when delivered in primary care settings (e.g., [12, 13]). Frequently primary care is the first and only contact individuals have with the health care system, and most people visit primary care at least once a year [14], making the primary care visit an opportunity to reach a population that may otherwise be untreated.

The sustainment of evidence-based practices after implementation support ends is an understudied area in health care. It is an important public health issue, as investments supporting the implementation of evidence-based practices are wasted if they are unable to be sustained following an initial implementation support period [15]. A review of the literature by Stirman et al. [16] concluded that the research on health care program sustainability is fragmented and underdeveloped. In general, implementation theories suggest that various external, internal, practice, and process-specific supports are needed for an organization to continue delivering evidence-based treatments after initial support ends (e.g., the Exploration, Preparation, Implementation, Sustainment (EPIS) model and the Consolidated Framework for Implementation Research (CFIR) [10, 17, 18]). External or “outer setting” supports refer to factors outside of the organization implementing the practice, such as the policy and fiscal environment as well as community support and aspects of the targeted patient population. Internal or “inner setting” supports refer to factors within the organization, such as leadership and staff backing, climate and culture, and internal resources to implement the practice. Also, elements of the evidence-based practice itself, such as its complexity, compatibility, and/or fit within the organization, are thought to impact sustainability. Finally, the process by which the evidence-based practice is adopted and implemented over time has also been identified as critical for continuation.

A growing number of empirical studies on the sustainability of evidence-based behavioral health treatment programs following the end of initial support have been conducted since the Stirman et al.’s [16] review. In general, the results from these studies are consistent with what implementation theory predicts, that is, factors related to the external, internal, practice and process are relevant to sustainability (e.g., [19–22]). Many studies also suggest that while elements of an evidence-based practice may be sustained, it is typical for some adaptation to occur whereby “partial” rather than “full” sustainment is more likely. For example, Aarons and colleagues [15, 23] found that many community-based sites continued to deliver a child neglect intervention following an initial implementation support period showing “operational” sustainment. However, the “structural” elements that help ensure quality delivery, such as ongoing coaching and supervision, were discontinued. These findings suggest that although an evidence-based treatment may be continued following the end of initial support, the fidelity may be compromised and inhibit provision of the outcomes achieved under more ideal conditions.

Recent empirical evidence supports the idea that multiple factors may be responsible for the continuation of substance use disorder treatment changes in routine practice settings [24, 25]. For example, our previous work has shown that four main factors were associated with sustainment: external setting characteristics (including funding stability and community partnerships); inner setting characteristics (including political support, organizational capacity, and clinical supervisor turnover rates); intervention characteristics (such as staff perceptions of the treatment’s complexity, relative advantage, and perceived success); and finally, the implementation process (i.e., the number of staff certified to deliver the treatment during the implementation period) [24, 25]. However, these studies were conducted in routine substance use treatment programs, not in primary care settings. Several factors unique to primary care could impact the sustainability of substance use disorder treatment. For example, primary care settings are more likely to have a physician on staff, which could positively impact the sustainability of medication-assisted treatment [26, 27]. However, given the core mission of the primary care setting is general health treatment rather than care for substance use, this could potentially have a negative impact on sustainment of treatment for these conditions. In a related study, Krist and colleagues [28] examined the adoption and continuation of an electronic substance use screening procedure among nine diverse primary care settings, including federally qualified health centers (FQHCs) in the US. They found that none of the primary care clinics continued the screening procedure as implemented during the research study, but six of the nine settings continued to implement certain elements of the screening. These findings suggest adaptation was necessary and “full sustainment” of the screening procedure delivered during the initial support period was not feasible.

This mixed methods study examines whether the extent to which AOUD treatment was sustained following the end of an implementation intervention in a large FQHC. FQHCs are community health clinics that receive support from the US government to provide primary care and other services to medically underserved populations. The study focused on AOUDs because these disorders are common among primary care patients and because there are effective, FDA-approved medications for use in medical settings [29–35]. More specifically, we examined treatment sustainment by examining AOUD care receipt and medicated-assisted prescribing behaviors over time to examine whether the treatment was continued at levels achieved during the implementation support period. Also, we used staff surveys to assess perceived treatment effectiveness and perceived compatibility, two characteristics theorized to be associated with implementation [18]. Finally, we conducted staff interviews and focus groups to identify facilitating and inhibiting factors to AOUD treatment sustainment. We anticipated that care would be continued, but that the delivery might be adapted to fit the resources available.

## Methods

### Study setting

The study was conducted in two large adult primary care clinics operated by a FQHC. The two clinics served over 22,000 low-income patients annually. Patients were racially and ethnically diverse, with 58% identifying as Latino/a, 26% identifying as White/Caucasian, 11% identifying as Black/African American, and 4% identifying as of Asian descent. RAND’s Institutional Review Board approved and monitored the study. A data sharing agreement between the FQHC and the research organization was used to gather information about treatment receipt.

### Study context

We used data from a study designed to examine the effect of a dual intervention implementation support strategy—an organizational readiness intervention and a collaborative care intervention—on the provision of AOUD treatment in primary care [36–38]. The organizational readiness intervention was launched prior to the collaborative care intervention; these two interventions have been previously described elsewhere (e.g., see [38, 39]). In brief, the organizational readiness intervention consisted of a number of implementation strategies designed to enhance the capacity of the organization to deliver AOUD care. These strategies included staff training in AOUD screening and treatment, developing and piloting the screening and treatment procedures, and conducting Plan-Do-Study-Act cycles to improve care delivery. The collaborative care treatment intervention included the utilization of a care coordinator, access to a six-session psychotherapy that incorporated motivational-interviewing and cognitive behavioral components, and access to medication-assisted treatment (extended-release injectable naltrexone (i.e., “Vivitrol”) for alcohol use disorders and buprenorphine/naloxone (i.e., “Suboxone”) for opioid use disorders). The staffing needed to provide the psychotherapy and medication-assisted treatment services were not funded by the research grant. Rather, staff already employed at the clinics were trained to incorporate these services into their existing job duties.

**Fig. 1:**
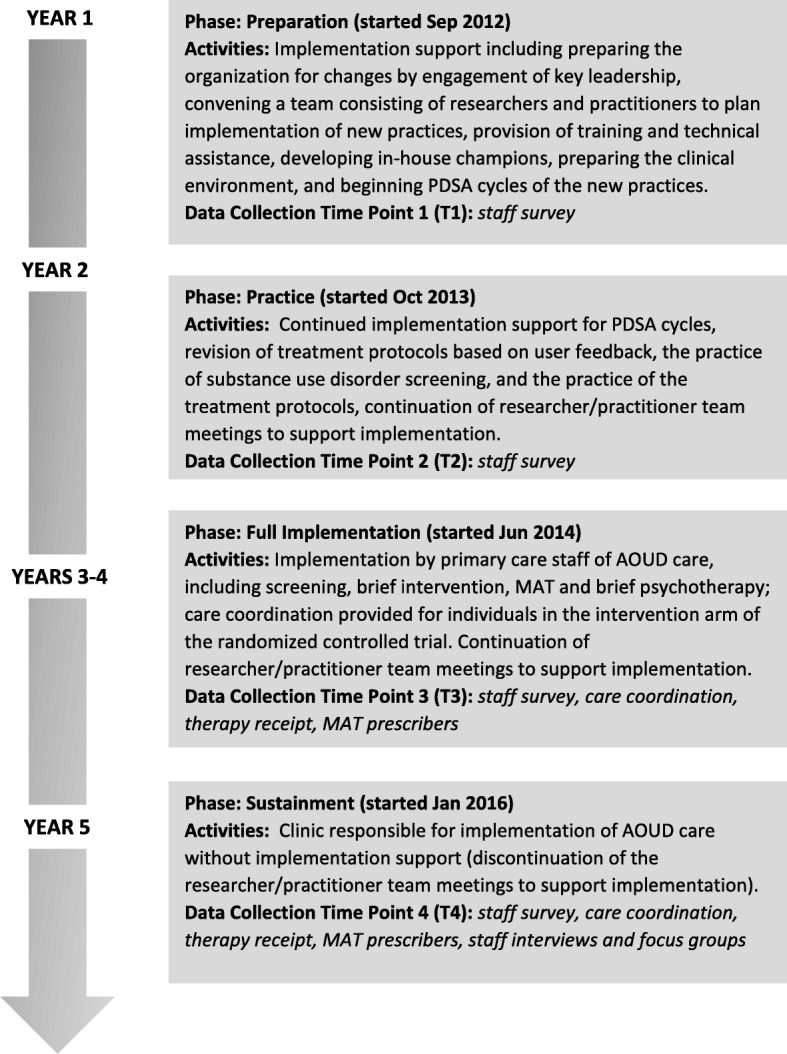
Implementation study timeline

We describe the study and data collection in four phases labeled “preparation,” “practice,” “full implementation,” and “sustainment” (see Fig. 1). During the preparation phase, researchers engaged key clinic leadership and documented the clinic workflow to prepare for the adoption of AOUDs screening procedures and the collaborative care treatment intervention. Existing clinic staff were trained to deliver these services. Practice refers to the study phase where the clinics piloted the screening and treatment (i.e., psychotherapy and medication assisted treatment) protocols. During this phase, researchers worked collaboratively with clinic staff to change the protocols based on initial testing and feedback. Together, these phases lasted approximately 2 years. Full implementation occurred during the randomized controlled trial (RCT) where patients who screened positive for an alcohol and/or opioid use disorder(s) were assigned to either the collaborative care intervention or usual care. This phase lasted approximately 2 years. The sustainment phase corresponded to approximately 1 year following the end of RCT enrollment period and 6 months after research staff ended implementation support. During this phase, support by the research staff was no longer available and the delivery of the intervention components (i.e., screening, therapy, and medication-assisted treatment) was determined by clinic staff.

### Participants

Participants were full-time administrative staff, medical and behavioral health providers, and general clinic staff, including medical assistants and discharge coordinators, front desk, call center, and security staff. All staff that fulfilled these positions at the two clinics were invited to participate in the study. The average age of participants was 44, and participants were mostly female (84%). Staff identified themselves most as Hispanic (70%) or White/Caucasian (23%). More than half (52%) had been in their current position at the clinic for more than 10 years.

### Measures and procedures

#### Treatment receipt and prescribing behaviors

We examined AOUD treatment receipt and prescribing behaviors among medical providers during two time periods, the implementation phase while the RCT was underway, that is, when all the procedures had been fully tested and executed (January–July 2016) and during the sustainment phase, that is, 1 year later following the end of the implementation phase (January–July 2017; see Fig. 1). To examine the receipt of care coordination [39] and AOUD-specific psychotherapy [40], we examined the number of unique patients who received at least one of each of those services during those time periods using information from the FQHC’s electronic database. We were unable to use patient-level data to monitor medication-assisted treatment delivery, because records of medication receipt were not adequately tracked for this purpose. More specifically, one medication was provided for free during the study and tracked on a study-specific log and, therefore, was not tracked consistently after the RCT ended. The other medication was not available through the clinic pharmacy and prescriptions were not always tracked in the electronic record. Therefore, we monitored medicated-assisted treatment at the provider level by examining the number of staff who prescribed it during the two study phases. This is consistent with Proctor et al.’s [41] definition of staff penetration, that is, we examined the percentage of medical providers who delivered the evidence-based practice (i.e., medication-assisted treatment for alcohol and/or opioid use) out of the number of medical providers eligible (i.e., employed) during the two study periods. Medical provider information was collected using a log we created that was completed by clinic personnel that gave each medical provider’s employment start and end dates, and prescribing behaviors during the implementation and sustainment phases.

#### Perceived treatment effectiveness and compatibility

A staff survey was conducted over the four study phases (see Fig. 1). We used four locally developed statements to assess staff agreement that “Substance use disorders can be effectively treated in primary care” to assess perceived treatment effectiveness at a general level; “Substance use disorders can effectively be treated at [clinic name]” to assess perceived treatment effectiveness at the clinic level; “Providing medications to patients with alcohol and opioid use disorders fits with [clinic name’s] mission and goals” to assess fit of the medication assisted treatment; and “Providing counseling to patients with alcohol and opioid use disorders fit with [clinic name’s] mission and goals” to assess fit of the psychotherapy. Response options ranged from “1” designated as “strongly disagree” to “5” designated as “strongly agree”.

#### Treatment sustainment facilitators and barriers

In concert with the survey time points, we conducted interviews and focus groups with clinic staff. We conducted one-on-one in-person interviews with key administrative staff, including the Chief Executive Officer, Chief Medical Officer, Associate Chief Medical Officer, Chief Operating Officer, Mental Health Director, Head of Nursing Staff, Front Desk Supervisor, Care Coordinator Supervisor, and Security Supervisor. We conducted two types of focus groups, one with medical providers (who could provide the medication-assisted treatment) and one with mental health providers (who could provide the psychotherapy). These focus groups were scheduled during regular staff meeting times to facilitate participation. Participation was voluntary. For this study, we utilized interview and focus group data collected at the fourth time point corresponding to the sustainment period. Information from the previous study periods is available in Storholm et al. [42].

Semi-structured protocol guides were used for both the interviews and focus groups. The guides first asked “grand tour” questions related to AOUD care, including screening, collaborative care, AOUD psychotherapy, and medication-assisted treatment (MAT). Specific probes were used to elicit more detailed responses about possible facilitators or barriers to sustaining AOUD care at their clinic. For example, staff were asked “Do you think that [clinic name] is going to continue to deliver the continuum of care for substance use disorder treatment, including screening, medication, and therapy?” and then follow-up questions such as “Can you describe why you think screening will be continued (or not continued)?” Respondents were also asked whether any changes were being made at the clinic to help continue treatment delivery and whether they saw any barriers to maintaining the AOUD treatment. Respondents were probed about screening, care coordination, medication and psychotherapy, if these topics were not spontaneously mentioned.

### Analyses plan

#### Treatment receipt and prescribing behaviors

Counts (i.e., number of unique patients) or proportions (i.e., providers prescribing out of providers eligible to prescribe) were calculated for each time point and examined descriptively to identify whether treatment delivery was similar or different across the full implementation and sustainment study phases.

#### Perceived treatment effectiveness and compatibility

Staff responses were aggregated across each survey time point. Next, group means and standard deviations were derived to describe an overall organizational perception rating for each time point. For each of the four items, the group mean value at the first time point was compared to the group mean value at the fourth time point to determine the level of change across time because our primary interest was to see whether there was significant change from the pre-intervention period to the sustainment period. Changes were assessed using *t* tests. We also examined and present graphically the group mean values at each survey time point to describe change over time.

#### Treatment sustainment facilitators and barriers

To identify staff perceptions of the sustainability of AOUD care in their clinic along with facilitators and barriers to sustainment, two trained research assistants reviewed interview and focus group transcripts and categorized any statements regarding the sustainability of AOUD care using a qualitative analysis software program [43]. Excerpts from the focus group transcripts were considered as equivalent to excerpts from the interview transcripts because we were unable to identify and quantify disparate respondents within the group. Excerpts regarding sustainability were then further evaluated and coded by the research assistants as to whether the content was related to external factors, internal factors, perceptions about the treatment or implementation processes, consistent with domains specified in the CFIR [18]. Next, one of the study researchers (Hunter) reviewed excerpts and independently coded them using the CFIR domains. Excerpts that were consistently coded across the coders and researcher were retained. The frequency of excerpts associated with the CFIR domains was identified. Themes that were mentioned at least three times in separate excerpts from respondents were flagged for reporting purposes.

## Results

### Treatment receipt and prescribing behaviors

#### Care coordination

The number of unique patients receiving care coordination increased from 33 during the full implementation period to 139 in the sustainment phase demonstrating over a fourfold increase.

#### AOUD psychotherapy

The number of unique patients receiving psychotherapy increased from 25 in the full implementation period to 75 in the sustainment phase, demonstrating a threefold increase.

#### Prescribing behaviors

The proportion of eligible providers prescribing medication for alcohol use was stable over time with a little over 80% at both time points. The proportion of eligible medical providers prescribing for opioid use disorders was lower at both time points than it was for alcohol use disorders, and decreased somewhat at the second-time point (from 56 to 45%) (Table 1).

**Table 1:** Number of patients receiving care and prescribing behaviors

	Implementation period (January–June 2016)	Sustainment period (January–June 2017)
Number of unique patients who received care coordination visit	33	139
Number of unique patients who received therapy for alcohol and/or opioid use	25	75
Percentage of medical providers prescribing MAT for alcohol use	83%	82%
Percentage of medical providers prescribing MAT for opioid use	56%	45%

### Perceived treatment effectiveness and compatibility

#### Staff survey response rates

Due to staff turnover and varying participation levels, the sample size changed over time. Response rates ranged from 74% at first time point (66 out of 102 staff) to 94% at the fourth time point (90 out of 96 staff). Response rates at the interim time points were 73% at the second time point (70 out of 96 staff) and 81% at the third time points (72 out of 89 staff).

**Fig. 2:**
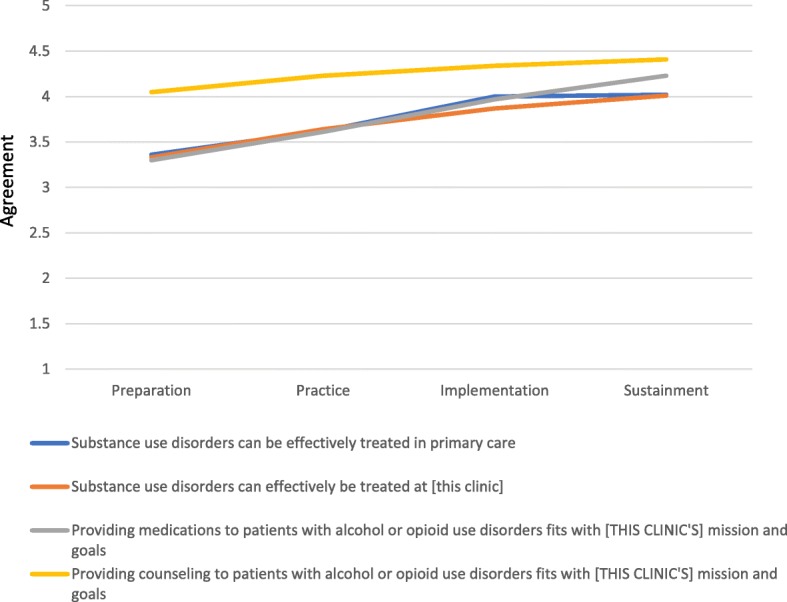
Staff perceptions of treatment effectiveness and compatibility over time

Group mean values across the four time points for each item are shown in Fig. 2. The overall pattern is consistent with the expectation that perceptions would improve over time as clinic staff became more familiar with and practiced substance use disorder care. Positive perceptions were maintained from the full implementation (i.e., time 3) to the sustainment (i.e., time 4) study phases.

#### Perceived effectiveness

The mean agreement rating for the statement that “substance use disorders can be effectively treated in primary care” was 3.36 (SD = 0.89) at the first time point and 4.02 (SD = 0.94) at the fourth time point, showing a statistically significant increase (*t* = 4.65; *p* < 0.001). Similarly, the mean agreement rating for the statement that “substance use disorders can effectively be treated at [this clinic]” was 3.33 (SD = 0.89) at the first time point and 4.07 (SD = 0.87) at the fourth-time point, demonstrating a statistically significant increase over time (*t* = 5.39; *p* < 0.001).

#### Compatibility

The mean agreement rating for the statement “providing medications to patients with alcohol and opioid use disorders fits with [this clinic’s] mission and goals” increased over time from 3.30 (SD = 0.99) at the first time point to 4.23 (SD = 1.01) at the fourth time point (*t* = 5.97; *p* < 0.001). Similarly, the mean agreement ratings for “providing counseling to the patients with alcohol and opioid use disorders fits with [this clinic’s] mission and goals” also improved over time from 4.05 (SD = 0.86) at the first time point to 4.41 (SD = 0.87) at the fourth time point (*t* = 2.67; *p* < 0.001). Of note, support for the provision of AOUD-specific psychotherapy at the clinic was higher than medication-assisted treatment at both the time points.

### Treatment sustainment facilitators and barriers

Excerpts from the interviews and focus groups were evaluated to identify the following: (1) whether staff perceived that AOUD treatment continued following the end of the implementation support phase; (2) what factors helped explain why it was or was not continued; and (3) what would be challenging to sustain and why. Participation rates were good. Individual interviews were conducted with representatives from the nine administrative positions that were targeted, for a 100% response rate. For the medical provider focus group, half of those eligible participated. For the mental health focus group, over 80% of those eligible participated. A total of 29 individuals participated in an interview or focus group. The main themes and illustrative excerpts are presented in the following section.

Overall, staff agreed that AOUD treatment was being continued. The factors mentioned by staff to help maintain it included perceived fit with the organization/clinic mission, e.g.,

I think it fits the model, the primary care model that we’ve envisioned for the future. I think particularly as I mentioned earlier with the way payment reform is going, both on a federal and a state level, we would be foolish not to move in that direction.

Another staff member reported:

And if we don’t care enough about our patients to treat that particular illness or their substance use issue, we don’t care about their health then. So the message to me is you either care about the whole person or you don’t care. So we have to treat. I mean, once you’ve taken off the blinders, which we’ve done, you can’t put them back on. We all see it now. You can’t put it back on.

Staff also mentioned that AOUD care had become institutionalized into the practice model at the clinics, e.g.,

You know I really do feel like the momentum is there, that it’s really become part of what we do. It’s hard to imagine, like, why we would undo that at this point in time.

Respondents also reported the importance of leadership support, the presence of program champion(s), and overall staff support, e.g.,

Dr. [name] is really good about disseminating that information and making sure that the staff is aware of what’s happening or not happening or which direction we’re going or what we need to do to sustain or do what we need to do with the patients.

Another interviewee reported:

I mean we were lucky to have some champions at the top when we first started, as well as to have ground folks always here with the MAs and the volunteers and kind of doing it from both ends.

We also asked about the sustainment of the different elements of the care model: screening, care coordination, psychotherapy, and medication-assisted treatment. In reference to screening, staff reported that it had become institutionalized and part of routine care at the clinic, e.g.,

I know the screening tool’s even been moved into the electronic system so it just comes up and the MAs (i.e., medical assistants) read the questions.

Another respondent reported:

I think the screening—everyone recognizes two things in the screening. A, it’s cheap. You’re already there with the patient so you ask them a few questions. And B, it’s critical to their health, so why wouldn’t we do it?

We did learn that while screening continued for AOUDs, the procedure had been adapted after the end of the full implementation (RCT) phase where instead of aiming to screen every patient at every visit, they instituted a protocol where patients were screened at 6-month intervals.

Regarding care coordination and psychotherapy, respondents told us that they had received additional funding since the research project ended to continue providing these components, including the funding of staff positions, e.g.,

Well, we got a grant to hire people, so we hired three new staff for it.

Staff noted that their psychotherapeutic approaches to treating substance use had expanded as a result of the implementation support, e.g.,

Of course. I mean, we were doing it before but we weren’t doing substance abuse-specific work. We were doing therapy with substance abusers, but now we’re trying to do the rest and we would never go back. Because you added information to our arsenal of techniques; we wouldn’t take them out.

Staff also commented that the psychotherapy provided to clients had become more structured due to the implementation support. However they were unsure whether the structured approach could be maintained over time, suggesting potential issues with ongoing fidelity, e.g.,

I think what’s interesting is it’s a more structured approach. And I kind of wonder because I think that is a new concept in mental health here. I feel like it’s been largely sort of whatever happens in therapy and not necessarily in a structured way. So I kind of wonder how that structure would be maintained through time. But then again, I don’t really know, but I could see that it might look a little differently through time. It depends on the leadership and how rigorous they may try to maintain that structure.

Staff training appeared to be a concern or potential barrier to continuing care due to staff turnover or other uncertainties, such as funding for positions, e.g.,

I think it could be easy for us to have that kind of approach to practice slip away if we’ve got enough kind of turnover.

Another staff member reported:

I think the training. We’ve talked about these, the mental health, do they have the capacity to continue it? I mean, I think there’re a lot of “ifs” on that too.

Comments regarding the continuation of medication-assisted treatment suggested that sustainment would be challenging due to perceptions that financial support was needed to purchase the medication. This concern was by far the most frequently mentioned challenge to continuing treatment, e.g.,

I know it’s very expensive and how do we sustain that cost. It’s always about money, right?

Another staff member reported:

I think the money is the big piece of the medications. I just don’t know how we’re going to—the Vivitrol. Why is the Vivitrol so expensive?

In sum, analyses of the qualitative data showed that participants expressed support for the continuation of AOUD treatment in their clinic. A new funding source allowed for the expansion of staffing to address it, but there were concerns expressed about whether the level of care would be maintained over time due to staff turnover and other uncertainties, such as funding and the potential for reimbursement for services. Finally, the most frequently reported barrier to continuing care was concern about funding for medication-assisted treatment.

## Discussion

In this study, we found that a large FQHC in the US continued to provide AOUD treatment after the end of implementation support. Regarding some elements of care, including care coordination and psychotherapy, organizational capacity increased following the loss of implementation support, suggesting that these aspects had become important components to the organization to maintain over time. Leadership support and the external context appeared to be the main drivers for these findings, given that opportunities were available and the organization applied and successfully received additional financial support to continue these elements after the implementation support ended. Consequently, those additional funds helped to provide staffing to expand services to individuals identified with an AOUD in their clinics. More specifically, the funds allowed the clinic to hire staff to provide care coordination and provide a full spectrum of behavioral treatments, including both group and individual therapy and case management. These additional funds were obtained without the direct assistance of the research team, that is, clinic leadership sought the funding without researcher support. These findings suggest that a multi-year implementation support approach led the FQHC staff to take advantage of changes in the external context that helped increase attention and treatment options available to address AOUDs in their setting.

Regarding staff perceptions, we found that primary care staff were supportive of the concept of providing care for AOUDs in their setting and these perceptions improved over time as the FQHC instituted practices as part of a multi-year implementation support effort. The staff reported that the AOUD treatment had become institutionalized and fit within the clinic’s philosophy and mission to treat the “whole person.” Given that clinic staff had learned how to address substance use, they stated that they did not perceive a reason to discontinue it following the end of implementation support.

However, it is also important to note that sustaining treatment for AOUDs into these primary care settings required adaptation. For example, the clinics modified the care model that reduced staff effort, for example, rather than screen at every visit, the organization shifted to a 6-month screening protocol. There was also suggestion that the structured elements of the AOUD psychotherapy might be discontinued over time which may negatively impact treatment fidelity and ultimately, outcomes. Clinic staff also expressed concern over sustainability, especially around two factors, staff turnover and the provision of medications. More specifically, respondents indicated that although current staff were trained to address substance use disorders, staff turnover could result in a lack of expertise without a plan in place so that new staff had the requisite skills or that the clinic had ongoing access to addiction training. Related to this, we found a slight reduction in the percentage of providers prescribing medications for opiate use, but not for alcohol use. We are unsure whether this reduction in prescribing behaviors for opiate use disorders will result in patient-level access problems because staff reported that patients eligible for opiate use medications may have been referred to prescribing physicians to help manage their care. Also, respondents reported that the medications were expensive and staff were unsure how patients could afford them in the long term. Thus, staff reported that they were not sure they felt comfortable promoting medication-assisted treatment if access to the medications could not be ensured. Of note, recent changes in reimbursement policies for the federally funded health care program (i.e., Medicaid) since this study ended may help to alleviate these concerns; however, these policies can change and may be region-specific making access dependent on local policies.

These findings are consistent with the studies in behavioral health (i.e., mental health and/or substance use) care settings which have found that multiple factors appear critical to the sustainment of evidence-based practice use [21, 23–25]. These studies indicate that a confluence of leadership and staff support are important, along with access to continued resources to maintain the practice under study. The significance of leadership and its impact on organizational culture and climate has been noted previously in the field (e.g., [44]). This study demonstrated that another vital leadership component is a proactive stance to obtaining external funding to continue practices following the loss of initial implementation support. Specific to the provision of AOUD treatment in primary care, additional training opportunities, especially in light of staff turnover, are an important element for long-term sustainment.

We also have evidence that external policies play an important role in practice sustainment. In this project, funding opportunities were available for the organization to continue to support AOUD care following the intervention period and policy changes that helped sustain access to medication-assisted treatment occurred. If these external factors were not present, it may have been very challenging for the clinic to continue or expand AOUD care, as observed in this study.

For other FQHCs or primary care organizations who may be interested in implementing or sustaining an AOUD program, we learned that ongoing funding and leadership support are critical to ensuring adequate organizational capacity (e.g., trained staff and access to medications) to support care. Implementing and sustaining an AOUD treatment program in primary care requires attention to multiple factors over time, both within and external to the organization, to ensure its longevity. For example, our research suggests that the following are needed: (1) a plan to train or hire staff with AOUD expertise when there is attrition; (2) the development of feasible protocols to identify and refer patients in need of AOUD treatment; and (3) long-term access to evidence-based care (e.g., medications and psychotherapy).

### Limitations

A few limitations to our study should be noted. An important component to the continuum of care is screening. Due to changes in screening procedures and to the electronic health record system over the study period, we were not able to capture accurate screening rates to compare across the study time points. The study is also limited in that it examined the experience at only two clinics operated by one FQHC. Results may differ in different settings and circumstances. We also may have missed input from staff that chose not to participate in the data collection activities. We also did not include qualitative information about sustainability planning that may occur in the preparation or implementation phases. Strengths include that we obtained feedback from multiple perspectives within the study setting, including administrative and front-line staff using both quantitative and qualitative approaches which led to rich and in-depth examination about the support needed to continue treatment for substance use disorders in primary care.

## Conclusions

This study informs the implementation science field about what factors are important for the sustainability of substance use disorder treatment provision within primary care clinics. Following an implementation research study, one large FQHC in the US sustained and, in some aspects, increased care for AOUDs. The number of unique patients who received care coordination and psychotherapy increased following the end of implementation support, likely due to the increased staffing to provide these services. Challenges remained, however, due to changes in screening protocols and concerns expressed over the provision of medication-assisted treatment. Multiple factors, both within and outside the organization, appeared to be related to the sustainment of care suggesting the importance of comprehensive approaches that target multiple levels to improve evidence-based practice use following the end of implementation support.

## Abbreviations

AOUD: Alcohol and/or opioid use disorder
CFIR: Consolidated Framework for Implementation Research
EPIS: Exploration, Preparation, Implementation, Sustainment
FDA: Food and Drug Administration
FQHC: Federally qualified health center
M: Mean
MAT: Medication-assisted treatment
NIDA: National Institute on Drug Abuse
RCT: Randomized controlled trial
SD: Standard deviation
US: United States
